# The Crosstalk Between Brain Energy Metabolism and Neuropathic Pain: Mechanisms and Therapeutic Implications

**DOI:** 10.3390/metabo15120755

**Published:** 2025-11-21

**Authors:** Jiangtao Wang, Baitong Liu, Jinyan Liu, Zhuoxi Hou, Guangxin Xie, Xiaoyi Xiong, Shuguang Yu

**Affiliations:** Acupuncture and Tuina School, Chengdu University of Traditional Chinese Medicine, Chengdu 610075, China; wangjttt958@163.com (J.W.); liubaitong@stu.cdutcm.edu.cn (B.L.); ljyczyzt559@stu.cdutcm.edu.cn (J.L.); houzhuoxi111@stu.cdutcm.edu.cn (Z.H.); xieguangxin@stu.cdutcm.edu.cn (G.X.)

**Keywords:** neuropathic pain, glucose metabolism, lipid metabolism, lactate metabolism, amino acid metabolism

## Abstract

Normal physiological brain activity relies on precise and orderly energy supply. The brain’s complex energy metabolism (encompassing glucose, lipid, lactate, and amino acid metabolic pathways) underpins neuronal function. Neuropathic pain severely impacts patients’ quality of life, and traditional therapies often prove ineffective. This condition is frequently accompanied by energy metabolism disorders in relevant brain regions. Dysregulation of metabolic pathways disrupts neuronal energy supply and signaling, impairs synaptic transmission, and triggers abnormal glial interactions and neuroinflammation, thereby driving the onset and chronic progression of neuropathic pain. This paper systematically elucidates the impact of metabolic pathway imbalances on neuropathic pain and explores potential therapeutic strategies targeting energy homeostasis. It aims to provide novel theoretical foundations and treatment approaches for the clinical management of neuropathic pain.

## 1. Introduction

Pain is an uncomfortable sensory and emotional experience linked to real or possible tissue damage [1]. Neuropathic pain, as defined by the International Association for the Study of Pain (IASP), is pain caused by lesions or diseases affecting the somatic sensory system, and it is a notable subtype of chronic pain [2]. Epidemiological research estimates the prevalence in the general population to range from 6.9% to 10% [3]. Compared to other chronic pain conditions, neuropathic pain is characterized by a protracted course and treatment resistance, significantly impacting patients’ disturbances, anxiety, and depression, and overall quality of life [4,5]. The etiology is complex. In addition to nerve compression, it is often associated with nerve damage caused by conditions such as diabetes, infection, or autoimmune disorders [6]. Despite some progress in research on neuropathic pain treatment, antidepressants and antiepileptic drugs remain the primary clinical treatment options, offering limited efficacy and significant side effects [7]. This compels us to explore novel pathophysiological pathways in pursuit of safer and more effective therapeutic interventions.

Neuropathic pain is not merely a simple abnormality in signal transmission; its onset and persistence are deeply causally linked to energy metabolism imbalances within the brain microenvironment. Research indicates that disrupted brain energy metabolism is a key driver in the generation and chronicity of pain, rather than merely a concomitant phenomenon [8]. As a highly energy-demanding organ, the brain relies on continuous, precise ATP supply for all physiological processes—including neuronal action potentials, synaptic transmission, and neurotransmitter synthesis and release [9,10]. Under neuropathic pain conditions, this delicate energy homeostasis is disrupted. Abnormal neuronal excitability and synaptic plasticity in pain-related brain regions (such as the primary somatosensory cortex and thalamus) inherently generate substantial energy demands [11,12,13]. Energy metabolism disruption further impairs inhibitory neuron function, exacerbating excitatory/inhibitory imbalance. Concurrently, it activates microglia and astrocytes, triggering persistent neuroinflammation. Under metabolic stress, microglia polarize toward a pro-inflammatory phenotype, releasing inflammatory mediators like IL-1β and TNF-α [14,15]. Astrocytes undergo a metabolic shift from mitochondrial oxidative phosphorylation to aerobic glycolysis, generating excessive lactate that sustains hyperalgesia via astrocyte-neuron lactate shuttling [16]. Thus, brain energy metabolism imbalance can be regarded as a core node integrating abnormal neuronal and glial activities, jointly driving the vicious progression of neuropathic pain.

This review systematically explores the mechanisms of brain energy metabolism in neuropathic pain, analyzing how variations and imbalances in the use of metabolic substrates such as glucose, lactate, lipids, and amino acids affect this condition. It also evaluates the translational potential of intervention strategies targeting these metabolic substrates or their key transport and oxidation pathways in alleviating neuropathic pain, offering new insights for clinical treatment approaches.

## 2. Literature Search Methods

For this review, literature searches were conducted across major international databases (PubMed, Web of Science, Embase) and Chinese databases (CNKI, Wanfang, VIP) to ensure comprehensive coverage of English and Chinese publications. The search period spanned from the inception of each database until October 2025, aiming to include both foundational and recent advances in the field. The search strategy employed core Medical Subject Headings (MeSH) terms such as “neuropathic pain” and “brain energy metabolism,” supplemented by iterative keyword refinement to enhance accuracy.

A total of 1465 records were initially identified. After removing duplicates and preliminary screening, 198 articles underwent full-text assessment. Literature selection prioritized thematic relevance, with inclusion criteria focusing on high-quality original research, systematic reviews, and key methodological papers addressing the relationship between brain energy metabolism and neuropathic pain. Studies were excluded if they did not involve a neuropathic pain context or focused solely on peripheral metabolism without addressing central regulatory mechanisms. Through this process, 91 publications met the criteria and were included in the review.

Additionally, manual screening of high-impact journals in the field was performed to identify potentially omitted studies. Although this review did not fully adhere to a standardized systematic review protocol, the combination of cross-database validation and a structured screening process ensures that the selected literature accurately and comprehensively reflects the mechanistic links between brain energy metabolism and neuropathic pain, thereby providing a solid evidence base for subsequent theoretical exploration.

## 3. Brain Energy Metabolic Characteristics

### 3.1. Glucose Metabolism

Adenosine triphosphate (ATP) is the primary energy currency in the central nervous system, essential for maintaining brain cell functions. Glucose generates ATP through metabolic processes, providing a continuous and controllable energy supply for brain physiological functions. Glucose enters the brain through glucose transporters (GLUTs), primarily GLUT1 and GLUT3, which are distributed according to cell type specificity [17]. GLUT1-55kDa is concentrated in blood–brain barrier endothelial cells, facilitating blood-to-brain transport, while GLUT1-45kDa is found in astrocytic foot processes, enabling interstitial-to-astrocyte glucose uptake. Neurons depend on the high-affinity, neuron-specific GLUT3 for glucose absorption [18,19,20,21].

Upon entering astrocytes, glucose is phosphorylated by hexokinase (HK) to form glucose-6-phosphate (G6P) [22], which is then directed into glycolysis, pentose phosphate pathway (PPP), and glycogen mobilization [23]. In the glycolytic pathway, G6P undergoes a multistep reaction to generate pyruvate, which can either enter the mitochondrial tricarboxylic acid cycle (TCA cycle) for oxidative energy supply or be converted to lactate during hypoxia or high metabolic demand and transported to the neuron via the lactate shuttle as its essential energy substrate. PPP accounts for less than 10% of the total glucose flux [23] and is essential for neuronal oxidative stress protection through the generation of NADPH and ribulose-5-phosphate, which provide cells with antioxidant defenses and nucleotide synthesis precursors. In the central nervous system (CNS), glycogen storage is exclusive to astrocytes [24]. When energy is sufficient, G6P is polymerized to glycogen by glycogen synthase [25]; when energy demand is elevated, glycogen phosphorylase rapidly breaks down glycogen back to G6P and re-enters glycolysis to maintain local energy homeostasis [26].

Unlike astrocytes, which are predominantly glycolytic, neurons are highly dependent on oxidative phosphorylation production capacity [27]. Glucose is phosphorylated to G6P, leading to pyruvate production via glycolysis, followed by TCA cycling and oxidative phosphorylation in the mitochondria to generate ATP for synaptic activity [28]. Approximately 10–20% of G6P is directed to the pentose phosphate pathway to produce NADPH, which sustains reduced glutathione (GSH) levels and neutralizes reactive oxygen species (ROS), thus safeguarding mitochondria from oxidative damage [24]. When oxidative phosphorylation is inhibited, neurons exhibit a limited capacity to rapidly upregulate glycolysis due to the cell-specific expression of relevant enzymes. Thus, maintaining PPP flux becomes a key compensatory mechanism for their resistance to oxidative stress [29].

### 3.2. Lactate Metabolism

Astrocyte glycolysis primarily generates brain lactate, which is increasingly acknowledged as a crucial energy source for neuronal activity [30,31]. The astrocyte-neuronal lactate shuttle (ANLS) hypothesis suggests that glutamate released at synapses activates astrocytes, leading to enhanced glucose uptake and lactate production; The lactate is then released into the extracellular space through monocarboxylic acid transporter protein 1/4 (MCT1/4), where it is absorbed by neuronal MCT2, converted to pyruvate, and enters the mitochondrial TCA cycle to support neuronal oxidative energy supply [32,33]. Functional studies further validated this model: glucose deprivation caused a 50% reduction in hippocampal synaptic transmission, while local glucose supply to astrocytes restored neural activity. Exogenous lactate mimicked this effect, and the MCT inhibitor α-CHCA blocked the rescue effect. This directly demonstrates that astrocytic networks generate lactate via glycolysis from glucose, which is then transported to neurons via MCTs for oxidative utilization [34]. Furthermore, pyruvate dehydrogenase (PDH) exhibits high activity in neurons but low activity in astrocytes, whereas the expression and activity of Pfkfb3, a key glycolytic regulator, are reduced in neurons but markedly elevated in astrocytes. This cell-specific expression profile results in active TCA cycling with limited glycolytic capacity in neurons, and active glycolysis with restricted pyruvate processing via the TCA cycle in astrocytes. Consequently, although glucose serves as an oxidative substrate in neurons, when both glucose and lactate are available, neurons may preferentially utilize lactate as a more efficient oxidative fuel [24,35,36].

### 3.3. Lipid Metabolism

In cases of hypoglycemia or disrupted glucose metabolism, the brain utilizes fatty acid oxidation and ketone body production as alternative energy sources to sustain normal function [37].

Astrocytes are the main site for fatty acid metabolism in the brain [38]. In response to stimuli, the blood–brain barrier and neurons generate excess fatty acids, which are then transported into astrocytes through specific transporters. There, they form lipid droplets and are stored within the endoplasmic reticulum [39]. Fatty acids support the TCA cycle and oxidative phosphorylation in astrocytes [40]. Astrocytes are the sole source of ketone bodies in the brain [37]. Excess fatty acids, coupled with decreased glucose uptake, undergo β-oxidation and enzymatic reactions to yield β-hydroxybutyrate (BHB) and acetoacetic acid [37,41]. Ketone bodies generated are transported from astrocytes to neurons via MCT1/2.Following a series of mitochondrial enzymatic reactions, they enter the TCA cycle to generate ATP for energy supply [42].

### 3.4. Amino Acid Metabolism

Multiple amino acids participate in brain functional activities and are closely associated with cerebral energy metabolism [24,43]. Glutamate, the main excitatory neurotransmitter in the central nervous system, is responsible for about 80% of the brain’s energy use through excitatory signaling [44]. Therefore, this section focuses on examining the role of glutamate metabolism in brain energy metabolism.

Glutamate plays a role in astrocyte-neuron energy metabolism through the glutamate-glutamine shuttle, essential for neurotransmitter balance and neuronal energy supply [45]. In astrocytes, glutamate is transformed into glutamine through the action of glutamine synthetase (GS), an enzyme uniquely found in these cells [46]. Released into the interstitial space through SNAT3/5, it is then absorbed by neurons. In neurons, glutamine is reconverted to glutamate by phosphate-activated glutaminase (PAG), a process known as the glutamate-glutamine cycle [47,48]. A study indicates that inhibiting glutamine synthesis in astrocytes disrupts neurotransmitter and energy homeostasis [49]. Glutamate functions as an energy substrate in brain metabolism. In astrocytes, glutamate may be a superior energy substrate compared to lactate, ketone bodies, or even glucose [42]. Aspartate transaminase (AAT) and glutamate dehydrogenase (GDH) are crucial in glutamate synthesis and oxidative metabolism. Both enzymes transform glutamate into α-ketoglutarate, a TCA cycle intermediate, with their specific roles potentially differing among cell types [48]. In astrocytes, GDH may dominate oxidative metabolism under high glutamate concentrations, while AAT is utilized in low-concentration environments [50]. In neurons, AAT is believed to play a more significant role in glutamate oxidation metabolism, which may result from a more active malate-aspartate shuttle within these cells [51].

**Figure 1:**
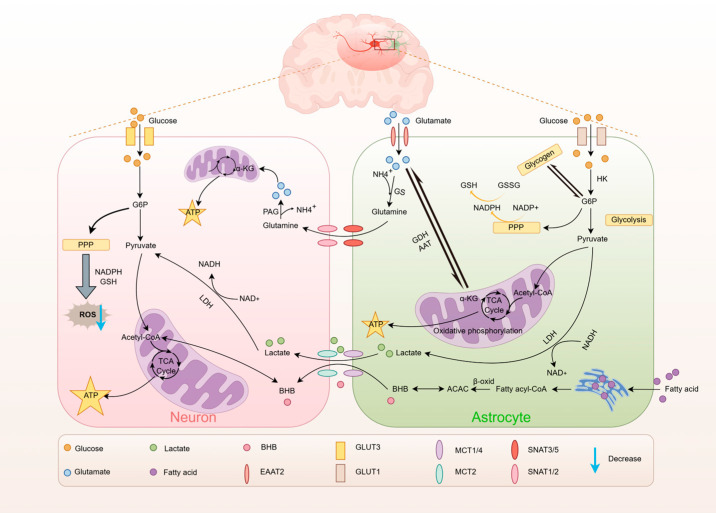
Physiological Processes of Brain Energy Metabolism. By Figdraw. HK (Hexokinase), G6P (Glucose-6-phosphate), PPP (Pentose phosphate pathway), TCA cycle (Tricarboxylic acid cycle), NADPH (Nicotinamide adenine dinucleotide phosphate), GSH (Glutathione), GSSG (Glutathione disulfide), LDH (Lactate dehydrogenase), NADH (Nicotinamide adenine dinucleotide), ATP (Adenosine triphosphate), α-KG (α-ketoglutarate), GDH (Glutamate dehydrogenase), ATA (Aspartate transaminase), GS (Glutamine synthetase), PAG (Phosphate-activated glutaminase), ACAC (Acetoacetate), BHB (β-hydroxybutyrate), ROS (Reactive oxygen species), EAAT2 (Excitatory amino acid transporter 2), GLUT1/3 (Glucose transporter1/3), MCT1/4/2 (Monocarboxylic acid transporter protein 1/4/2), SNATs (sodium-coupled neutral amino acid transporters), SNAT3/5 (System N), SNAT 1/2 (System A).

In summary, the complex metabolic activities of the brain form the foundation for neuronal function, and balanced, orderly brain energy metabolism provides the material basis for life processes (Figure 1).

## 4. Imbalance in Brain Energy Metabolism and Neuropathic Pain

**Figure 2:**
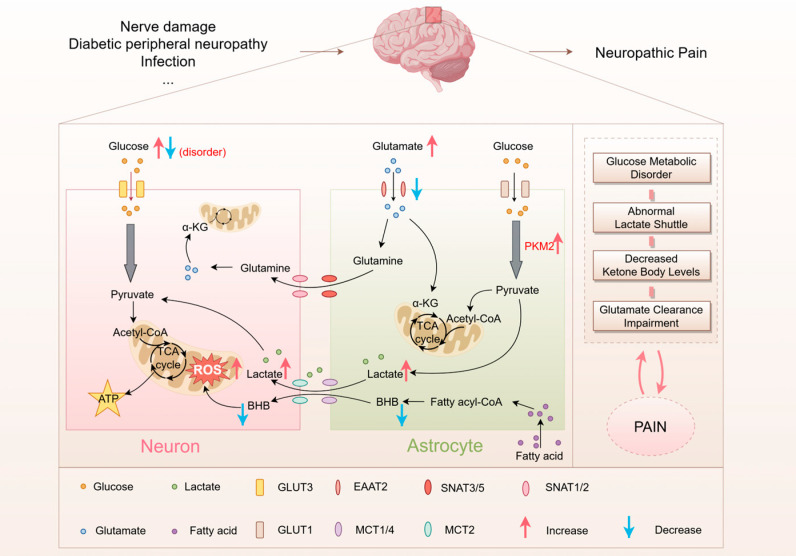
Neuropathic pain and cerebral metabolic disorders form a vicious cycle. By Figdraw. PKM2 (Pyruvate kinase M2).

Neuropathic pain and brain energy metabolism imbalance form a bidirectional vicious cycle: on one hand, persistent abnormal neuronal discharge and excessive glial cell activation lead to a surge in energy demand, triggering mitochondrial dysfunction, lactic acid accumulation, and reactive oxygen species bursts, which exacerbate synaptic plasticity abnormalities (such as central sensitization); on the other hand, glucose metabolism disorders and energy reserve depletion in key brain regions further impair pain regulation circuit function, perpetuating pain chronicity. Therefore, targeting energy homeostasis may represent a breakthrough therapeutic strategy for neuropathic pain (Figure 2).

### 4.1. Neuropathic Pain and Impaired Glucose Metabolism

Glucose is the primary energy substrate for the brain, and the stability of glucose metabolism to some extent reflects whether brain functional activity is normal [52]. Clinical studies have shown significant evidence that disruptions in cerebral glucose metabolism are linked to the persistence and development of neuropathic pain. A study revealed that patients with chronic neuropathic pain from brachial plexus avulsion (BPA) showed notably decreased glucose metabolism in the ipsilateral thalamus and S1 compared to healthy adults. Glucose metabolism was notably increased in the ipsilateral orbitofrontal cortex, contralateral insular cortex, and dorsolateral prefrontal cortex [53]. Research on cerebral metabolic alterations in patients experiencing pain after hemorrhagic pontine stroke identified decreased glucose metabolism in the contralateral angular gyrus and ipsilateral supplementary motor cortex. Furthermore, this reduction in glucose metabolism correlated with increased pain intensity [54].

In animal models, we observed that in right-sided BPA-treated mice, the experimental group exhibited elevated standard glucose metabolic activity in both the right and left thalamus compared to control mice [11]. In rats with the Spinal Neuronal Injury (SNI) model, glucose metabolism increased in the contralateral S1 posterior limb area, bilateral anterior insular cortex, thalamus, and cerebellar vermis; glucose metabolism decreased in the contralateral amygdala, bilateral splenoparietal cortex, prefrontal cortex, and hippocampus [12,55,56]. In the tibial and sural nerve transection (TST) model rats, an increase in metabolic activity was noted in the contralateral primary motor and sensory cortices during the first two weeks post-modeling. From the third week onward, there was an increase in activity in the central nucleus of the inferior colliculus and most of the cerebellum, accompanied by a decrease in activity in the periventricular gray matter and the primary and secondary motor cortices. This trend continued until the experiment concluded in the eighth week [57].

Neuropathic pain is associated with imbalances in glucose metabolism across various brain regions, each displaying unique abnormalities. This could be linked to the development of neuropathic pain.

### 4.2. Neuropathic Pain and Impaired Lactate Metabolism

Lactate is a key energy substrate involved in central sensitization [58], mediating pain generation and maintenance across multiple pain models [59]. Normal cells mainly depend on mitochondrial oxidative phosphorylation for energy, whereas cancer cells can perform aerobic glycolysis even in the presence of oxygen, known as the ‘Warburg effect’ [60]. In neuropathic pain, neuroinflammatory stimuli reprogram cellular metabolism, enhancing glycolytic activity and producing a similar effect [8]. Intracerebral lactate is primarily generated by astrocytic glycolysis. Consequently, impaired lactate metabolism in the brain manifests mainly through enhanced astrocytic glycolysis and abnormal ANLS.

Pyruvate kinase (PK) is an essential glycolytic enzyme that facilitates the transformation of phosphoenolpyruvate into pyruvate. PKM2 is a major isoform within the PK family, expressed in both normal and cancer cells [61]. Research indicates that peripheral nerve injury induces PKM2 upregulation via the ERK/STAT3 pathway [62]. Nuclear dimeric PKM2 interacts with hypoxia-inducible factor 1 (HIF-1), enhancing the expression of glycolytic genes such as GLUT1, SLC2A1, LDHA, and PDK1, which leads to increased glucose consumption and lactate production [14,63]. Concurrently, glutamate accumulated at synapses following chronic nerve injury enters astrocytes, activating membrane-bound Na^+^-K^+^-ATPase to promote glucose uptake and induce glycolysis. The resulting lactate shuttles back into neurons for energy supply [64,65]. ANLS represents a crucial supplementary energy source for neurons. In one study, intrathecal lactate injection significantly lowered the mechanical pain threshold in a neuropathic pain mouse model, whereas injection of astrocyte inhibitors/lactate dehydrogenase inhibitors/MCT inhibitors effectively reversed this effect. This demonstrates that activated astrocytes sustain neuropathic pain by supplying excessive lactate through abnormal lactate shuttling [16].

Based on the above research, improving the glycolytic pathway and lactate shuttle to correct lactate metabolism imbalance may offer new strategies for addressing neuropathic pain.

### 4.3. Neuropathic Pain and Impaired Lipid Metabolism

Lipids serve as crucial energy substrates for brain metabolic activity. Among these, BHB and acetoacetic acid not only function as alternative fuels sustaining brain and peripheral tissue function but also exert neuroprotective effects [66]. Studies indicate that neuropathic pain from chronic constriction injury (CCI) in mice leads to significantly lower BHB levels, whereas a ketogenic diet (KD) mitigates CCI-induced mechanical allodynia and thermal hyperalgesia, and reduces spinal neuroinflammation and microglial hyperactivation [67]. A study demonstrated that a single intraperitoneal injection of BHB significantly alleviated CCI-induced tactile allodynia, likely through the modulation of hydroxyl carboxylic acid receptor type 2 (HCAR2) [68]. Notably, ROS production occurs during mitochondrial oxidative phosphorylation, and excessive ROS-induced oxidative stress is a major contributor to pain. Chemotherapy-induced painful peripheral neuropathy causes a metabolic shift from oxidative phosphorylation to glycolysis, resulting in mitochondrial dysfunction and ROS accumulation [69]. The KD has been shown to enhance mitochondrial respiration and reduce ROS in mouse models [70]. Ketone bodies may alleviate pain by enhancing mitochondrial function and reducing oxidative stress.

Overall, lipid levels—particularly ketone bodies—are suppressed in neuropathic pain models, a finding closely linked to pain sensitization. Supplementing ketone body levels, such as through a ketogenic diet, may emerge as a novel therapeutic strategy for neuropathic pain.

### 4.4. Neuropathic Pain and Impaired Glutamate Metabolism

Glutamate, the main excitatory neurotransmitter in the central nervous system, is integral to neuronal excitation and synaptic transmission. Elevated extracellular glutamate levels can lead to excitotoxicity and neuronal death [71,72]. Studies suggest that increased cortical glutamate levels may play a role in central sensitization associated with neuropathic pain [73]. Clinical studies have demonstrated that under painful conditions, glutamate levels in the cortex, cingulate cortex, and thalamus are significantly elevated [74,75]. Therefore, excessive glutamate is associated with the maintenance and progression of neuropathic pain.

Excitatory amino acid transporters (EAATs) in neurons and glial cells are crucial for glutamate clearance, ensuring proper signaling and preventing excitotoxicity from extracellular glutamate buildup [76]. Changes in EAAT expression and function impair glutamate uptake and release, causing neuronal signaling dysfunction linked to neuropathic pain pathogenesis. EAAT2 is the main glutamate transporter in the central nervous system, responsible for about 95% of glutamate transport activity and essential for maintaining glutamate homeostasis [77,78]. Research shows that in rat models with SNI/CCI-induced neuropathic pain, levels of EAAT2 and glutamate/aspartate transporter (GLAST) are notably decreased. Electroacupuncture treatment effectively reverses this phenomenon, thereby alleviating pain [79,80].

Following nerve injury, glutamate transporters such as EAAT2 exhibit downregulated expression, leading to reduced glutamate uptake. Consequently, this increases glutamate levels in the synaptic cleft, leading to disrupted glutamate signaling. Understanding the mechanisms by which glutamate transporters regulate glutamate levels in nociception could offer new therapeutic targets for neuropathic pain.

### 4.5. Neuropathic Pain and Microglial Metabolism

In addition to astrocytes, microglia—the primary immune cells of the central nervous system—also play a critical role in the development and maintenance of neuropathic pain. The metabolic state of microglia is closely linked to their functional phenotype: under resting conditions, they primarily rely on oxidative phosphorylation to support surveillance functions; upon activation, however, they undergo metabolic reprogramming toward glycolysis to rapidly generate energy and biosynthetic precursors, thereby facilitating pro-inflammatory responses [64,81,82].

Studies in a CCI model have shown that activated microglia exhibit enhanced glycolysis, characterized by upregulation of key glycolytic proteins such as HIF-1α, PKM2, and GLUT1, along with increased lactate production. This metabolic shift not only promotes polarization toward a pro-inflammatory (M1) phenotype, leading to the release of cytokines such as IL-1β and TNF-α, but also amplifies neuroinflammation and synaptic plasticity changes, thereby exacerbating pain signaling [14,15]. Furthermore, enhanced glycolysis in microglia is associated with mitochondrial dysfunction and accumulation of reactive oxygen species (ROS), which may further contribute to pain chronicity [83]. Therefore, modulating microglial metabolism (particularly by inhibiting aberrant glycolytic activation) represents a promising novel strategy for alleviating neuropathic pain.

The above studies indicate that imbalances in brain energy metabolism are closely associated with the onset and progression of neuropathic pain. Understanding the disruption of different metabolic substrates in neuropathic pain may be beneficial for targeting and regulating metabolic disorders (Table 1).

## 5. Discussion

Imbalances in brain energy metabolism exhibit a complex bidirectional relationship with neuropathic pain. Under neuropathic pain conditions, glucose metabolism disorders manifest as distinct metabolic states across different brain regions, with these variations potentially linked to the pathogenesis of neuropathic pain. Regarding lactate metabolism imbalance, abnormal glycolysis in glial cells leads to excessive lactate accumulation, sustaining neuronal hyperexcitability through abnormal lactate shuttling mechanisms. Lipid metabolism imbalance in neuropathic pain primarily manifests as reduced ketone body levels. Glutamate metabolism imbalance may sustain pain through central sensitization mechanisms. Under neuropathic pain conditions, alterations in glutamate transporter expression and function disrupt glutamate uptake and release, subsequently triggering neuronal signaling disorders. Therefore, correcting brain energy metabolism imbalance is crucial for breaking the vicious cycle of energy disorder-pain and alleviating neuropathic pain, potentially offering new insights for future therapeutic strategies.

We aim to achieve relief from neuropathic pain by precisely modulating the balance of relevant metabolic substrates through targeted pharmacological approaches. In one study, gabapentin injection in SNI-induced neuropathic pain model rats significantly alleviated pain symptoms and inhibited glucose metabolism in the medial prefrontal cortex [55]. It has been demonstrated that dexmedetomidine exerts analgesic effects and can suppress glycolysis in neuropathic pain model rats by downregulating the expression of glucose transporters and key glycolytic enzymes, thereby reducing lactate levels in vivo [84]. Another study found that the MCT inhibitor 4-CIN and the LDH inhibitor genistein effectively reversed abnormal mechanical nociceptive thresholds in neuropathic pain model mice. This confirms that mechanical allodynia in neuropathic pain is driven by excessive L-lactate supplied by the abnormal ANLS, and that reducing lactate levels can effectively alleviate pain [16]. Ceftriaxone increases GLT-1 levels; administration of ceftriaxone in SNI model rats enhanced glutamate uptake, decreased extracellular glutamate concentrations, and thus relieved pain [85]. We believe that maintaining glutamate balance may represent the most promising target for future clinical interventions. Enhancing the expression or function of glutamate transporters (particularly EAAT2) to restore glutamate homeostasis is a highly promising non-opioid strategy that directly targets central pain mechanisms, avoids addiction risk, and has been validated in multiple neuropathic pain models. Drugs that modulate EAATs to maintain glutamate balance, such as ceftriaxone [86], clavulanic acid [87], and minocycline [88], have already entered early-stage clinical trials for neuropathic pain. Moreover, emerging compounds that allosterically modulate EAAT2 have shown neuroprotective properties in basic research [89], strengthening our confidence in this target. However, given that existing single-target drugs may still carry off-target risks and impose high economic burdens on patients, acupuncture—a non-pharmacological, multi-target therapy—warrants exploration. Studies indicate that electroacupuncture can effectively reverse CCI model-induced mechanical allodynia and elevated glucose metabolism in the medial prefrontal cortex of rats [90]. In type 2 diabetic peripheral neuropathy (T2DPN) model rats, we found that electroacupuncture improves oxidative stress, reduces lactate levels, and thereby restores metabolic control and ameliorates nerve injury [91]. In CCI model rats, electroacupuncture upregulates glutamate transporter levels and reduces glutamate concentrations, resulting in analgesic effects [79].

However, targeting brain energy metabolism for the treatment of neuropathic pain has certain limitations. Different neuropathic pain models (e.g., SNI, CCI, BPA) exhibit significant and sometimes contradictory patterns of cerebral metabolic alterations. This heterogeneity likely stems from differences in the type and location of the initial nerve injury, which activate distinct neural circuits and molecular mechanisms, ultimately shaping unique brain metabolic profiles. Therefore, the generalizability of metabolically targeted interventions validated in one model requires rigorous evaluation across multiple models. Most current strategies targeting energy metabolism are based on animal models, which typically involve highly standardized surgeries inducing single-etiology pain in genetically homogeneous young male animals. In contrast, human neuropathic pain has highly complex etiologies (e.g., diabetes, trauma) and exhibits substantial interindividual variability. Animal models also fail to fully replicate comorbid conditions such as anxiety, depression, and cognitive impairments often present in human chronic pain, each of which can independently influence brain energy metabolism. Consequently, metabolic mechanisms of pain identified in animals may be confounded in the complex human brain during clinical translation.

In summary, brain energy metabolism imbalance plays an important role in the pathogenesis of neuropathic pain. Future studies should further explore more targeted drugs and multi-target therapies for its treatment.

## 6. Conclusions

Disruption of brain energy metabolism serves as a key driver in the chronicization of neuropathic pain. Increased energy demands resulting from neuronal hyperexcitability and glial activation lead to region-specific disturbances in glucose metabolism, aberrant lactate production and shuttling, decreased ketone body levels, and impaired glutamate clearance. These metabolic alterations exacerbate central sensitization, oxidative stress, and excitotoxicity, which further disrupt pain-regulatory circuits and create a vicious cycle. As the mechanisms underlying substrate metabolism continue to be elucidated, targeting metabolic homeostasis may offer a novel pathophysiological foundation for the clinical treatment of neuropathic pain.

## Figures and Tables

**Table 1:** Changes in Various Metabolic Substrates in Neuropathic Pain. BPA (brachial plexus avulsion), SNI (Spinal Neuronal Injury), TST (tibial and sural nerve transection), CCI (chronic constriction injury).

Neuropathic Pain Model	Research Object	Type of Metabolic Substrate	Change	Reference
BPA	Human	Glucose	Decreased glucose metabolism in the ipsilateral thalamus and S1; increased in the ipsilateral orbitofrontal cortex, contralateral insular cortex, and dorsolateral prefrontal cortex	[53]
Pain after hemorrhagic pontine stroke	Human	Glucose	Decreased glucose metabolism in the contralateral angular gyrus and ipsilateral supplementary motor cortex	[54]
BPA	Mouse	Glucose	The experimental group exhibited elevated standard glucose metabolic activity in both the right and left thalamus compared to control mice	[11]
SNI	Rat	Glucose	Glucose metabolism increased in the contralateral S1 posterior limb area, bilateral anterior insular cortex, thalamus, and cerebellar vermis; glucose metabolism decreased in the contralateral amygdala, bilateral splenoparietal cortex, prefrontal cortex, and hippocampus	[12,55,56]
TST	Rat	Glucose	1–2 weeks: increase in metabolic activity was noted in the contralateral primary motor and sensory cortices 3–8 weeks: increase in activity in the central nucleus of the inferior colliculus and most of the cerebellum, accompanied by a decrease in activity in the periventricular gray matter and the primary and secondary motor cortices.	[57]
CCI	Rat	Lactate	Increased lactate levels	[14,15,62]
CCI	Mouse	BHB	Decreased BHB content	[67,68]
CCI	Rat	Glutamate	GLAST expression decreased, Glutamate levels increased	[79]
SNI	Rat	Glutamate	Glutamate levels increased	[80]

## Data Availability

This review article is based on previously published studies. All data analyzed in this work are publicly available through the cited references. No new datasets were generated or analyzed during this review. Links to the original sources are provided in the reference list.
